# Spatial Insights for Understanding Colorectal Cancer Screening in Disproportionately Affected Populations, Central Texas, 2019

**DOI:** 10.5888/pcd18.200362

**Published:** 2021-03-04

**Authors:** F. Benjamin Zhan, Niaz Morshed, Nicole Kluz, Bretta Candelaria, Eda Baykal-Caglar, Anjum Khurshid, Michael P. Pignone

**Affiliations:** 1 LiveStrong Cancer Institutes, Dell Medical School, University of Texas, Austin, Texas; 2 Texas Center for Geographic Information Science, Department of Geography, Texas State University, San Marcos, Texas; 3 Departments of Internal Medicine and Population Health, University of Texas Dell Medical School, Austin, Texas; 4 CommUnityCare, Federally Qualified Health Centers, Austin, Texas

## Abstract

**Introduction:**

Colorectal cancer (CRC) screening can reduce morbidity and mortality; however, important disparities exist in CRC uptake. Our study examines the associations of distance to care and frequency of using primary care and screening.

**Methods:**

To examine the distribution of screening geographically and according to several demographic features, we used individual patient-level data, dated September 30, 2018, from a large urban safety-net health system in Central Texas. We used spatial cluster analysis and logistic regression adjusted for age, race, sex, socioeconomic status, and health insurance status.

**Results:**

We obtained screening status data for 13,079 age-eligible patients from the health system’s electronic medical records. Of those eligible, 55.1% were female, and 55.9% identified as Hispanic. Mean age was 58.1 years. Patients residing more than 20 miles from one of the system’s primary care clinics was associated with lower screening rates (odds ratio [OR], 0.63; 95% CI, 0.43−0.93). Patients with higher screening rates included those who had a greater number of primary care–related (nonspecialty) visits within 1 year (OR, 6.90; 95% CI, 6.04−7.88) and those who were part of the county-level medical assistance program (OR, 1.61; 95% CI, 1.40−1.84). Spatial analysis identified an area where the level of CRC screening was particularly low.

**Conclusion:**

Distance to primary care and use of primary care were associated with screening. Priorities in targeted interventions should include identifying and inviting patients with limited care engagements.

**Summary**

**What is already known on this topic?**

Not much is known about factors associated with colorectal cancer screening rates among low-income populations.

**What is added by this report?**

Based on geocoded patient-level data in Central Texas, a large urban safety-net health system, our study suggests that patients residing less than 20 miles from primary care and screening facilities who regularly visit a primary care physician and have health insurance are positively associated with high rates of screening uptake.

**What are the implications for public health practice?**

Programs that are focused on increasing colorectal cancer screening among low-income populations can be more effective by providing assistance, such as mailed stool testing, to patients who live far from primary care and screening facilities and to those who do not regularly visit a primary care physician.

## Introduction

Colorectal cancer (CRC) screening is effective but underused, especially in medically underserved and disproportionately affected populations. Demographic factors and access to care variables are associated with screening (1–6). Although many factors are widely understood, including the importance of having health insurance and a regular source of care, the association between geography and cancer screening has been partially explored, and cancer screening in urban environments has not been well described. Spatial insights from geographic associations might help promote greater understanding of how different factors affect CRC screening, particularly among populations that are highly affected and may be more challenged by geographic barriers because of limitations in transportation and other competing demands (7–10). As such, spatial insights might be used to enhance interventions designed to overcome screening barriers.

Spatial insights are patterns related to a set of location-based observations and factors associated with the patterns. Spatial insights may provide novel information beyond commonly measured predictors of screening, such as insurance status or race/ethnicity. Furthermore, insights into patterns and factors may provide actionable information at the patient level to directly support the enhancement and implementation of effective interventions. Our study aimed to provide insights and help enhance efforts focused on increasing CRC screening rates in underserved populations of a large urban safety-net health system in Central Texas. Our primary objective was to determine the factors that are significantly associated with up-to-date CRC screening. Our secondary objective was to identify geographic areas where screening levels of patients are significantly lower in this safety net-health system.

## Methods

We used electronic health records, supplemented by additional geographic information, to examine CRC screening in an urban setting. The Office of Research Support and Compliance at the University of Texas at Austin and at Texas State University approved the institutional review board application for this study, and a waiver of informed consent was granted for the use of de-identified patient data (Box).

**Box. Data Sets Used in Spatial Insights for Understanding Colorectal Cancer Screening, 2019**

**Table:** 

Data set	Source	Variables in analyses
CommUnityCare (CUC) patient data	Data Core, The University of Texas, Dell Medical School	Resident addresses Race Ethnicity Age Medical home Sex CRC screening status Date of screening Financial class Primary care physician Number of primary care–related visits in 1-year
CUC clinics	CUC Health Centers	Clinic names Clinic addresses
Census tract-level data	US Census Bureau	TIGER/Line Shapefiles
Road networks	Environmental Systems Research Institute (ESRI)	Shapefile

The study was conducted in a large urban county in Central Texas among patients of a large federally qualified health center (FQHC) system. The system had approximately 100 providers serving nearly 98,000 patients in 2018 and provided care at 21 clinics that year. Because we were interested in average-risk screening, we studied adults older than 49 as of September 30, 2018. We defined the CRC screening status of a patient as either screened or unscreened as of September 30, 2018, based on records of having a stool test within the previous year or a colonoscopy within the previous 10 years. We extracted these data from patient records. We used FQHC system databases to collect patient information on several demographic variables and used other relevant databases for the spatial analysis.

Health insurance status indicated sources of financial support for health care, such as Medicaid, Medicare, private insurance, the Medical Access Program (MAP), grants for health care, or unknown (information not available). The MAP is a local program in Travis County provided by Central Health that covers medical care for qualifying Travis County residents. Patients with MAP benefits have low incomes, are ineligible for or not enrolled in Medicare or Medicaid, and are not covered by private insurance. Medical home was defined as the clinic where the patient received primary care and point of contact for CRC screening. Typically, Dell Seton Medical Center (DSMC) was the only site where uninsured patients were referred for colonoscopies and was also the main site of referral for health-insured patients in this system.

The initial patient data set contained 27,285 records. We obtained geographic locations of individual patients, based on their residential addresses, through geocoding. The locations of the 21 medical homes of the CommUnityCare patients and the DSMC were also geocoded based on the addresses of these entities. We used the geocoding tool in ArcGIS (ESRI) to perform geocoding (11). Among the 27,285 records, 3,843 cases (14.1%) were excluded from geocoding because of incomplete, insufficient, or incorrect addresses during initial examination of the records. In addition, 1,519 cases (5.6%) could not be included in geocoding because of incomplete or incorrect addresses. Some patients were homeless and did not have addresses on file. A total of 21,923 CommUnityCare patients were geocoded to street level to produce the geocoded patient data set that yielded an overall geocoding rate of 80.4%. All coordinates used in the analysis were residence locations at street level, not at the zip code polygon or any other areal unit level.

We then prepared 2 data sets for analysis. Data set 1 contained the 21,923 patient records. This data set was used for spatial analysis and mapping. Data set 2 consisted of 13,079 patient records with complete aspatial and spatial data for all needed variables used in logistic regression analysis. Aspatial data is information that is not related to location. This second data set included data only for non-Hispanic White, Hispanic, and African American patients.

When preparing data set 2, we began with the 21,923 records of patients with geocoded residence locations and excluded 8,844 (40.3% of 21,923) records to obtain the 13,079 records. Among these 8,844 records, 5,388 (24.6% of 21,923) records did not have information needed to accurately determine the driving distances from the patient locations to their medical homes because some patients were served by mobile medical facilities, and information about mobile facilities was not available. We defined driving distance as the shortest distance between the patient residence and the location of the health care facility in question. A total of 2,593 patients (11.8%) either did not have complete information about race/ethnicity or were categorized into population groups other than White, Hispanic, or African American; 371 (1.7%) had no information about sex; 376 (1.7%) had no information on health insurance status, and 116 (0.5%) were older than 75.

### Logistic regression analysis

We used logistic regression to examine how various individual and spatial factors were associated with up-to-date CRC screening. Other analyses were performed by using only aspatial variables to examine whether findings for the constrained population of 13,079 differed from those of the larger population of 27,285. Information is included to distinguish patients supported by the MAP or partially covered based on a sliding income scale. Logistic regression analysis was also performed using the larger data set. Results of these additional analyses confirmed results from the 13,079 data set.

### Spatial cluster analysis

To achieve the second objective of our study, we used spatial cluster analysis to determine whether significant concentrations of patients existed without up-to-date CRC screening. We used SaTScan version 9.6 (SaTScan) to perform the cluster analysis. This tool is based on the Spatial Scan statistic developed by Kulldorff (12), initially distributed by the National Cancer Institute. To avoid statistical bias, we followed standard practice and used the maximum allowable cluster size covering 50% of the total patients in the study area (13). Maximum allowable cluster size avoids the use of a predetermined cluster size, and therefore, helps detect any cluster size smaller than an area covering up to about 50% of the geocoded patients in this study. We used the Poisson probability model and performed 3 separate cluster analyses using residence locations of each patient for all patients combined, Hispanic patients only, and African American patients only.

## Results

Complete spatial and aspatial data were available for the analyses of the 13,079 patients. Among these patients, the overall up-to-date screening rate was 33.9%, and mean age was 58.1. Slightly more than one-half (55.1%) were female, and 56.0% identified as Hispanic, and most had MAP benefits. For the 27,285 patients in the initial patient data set, the overall up-to-date screening rate was 30.8%, and the rate among the 21,923 patients with geocoded residence locations was 32. 0%. Rates for other categories among the 27,285 patients and the 21,923 patients were similar. These categories include race/ethnicity, age group, sex, health insurance status, number of primary care–related visits in 1 year, spatial access to a medical home, and spatial access to the DSMC (Table 1).

**Table 1:** Characteristics of Patients in Colorectal Cancer Screening Study (N = 13,079), Central Texas, 2018

Characteristics	No. Patients (%)
**Race/ethnicity**	
Non-Hispanic White	3,194 (24.4)
Non-Hispanic African American	2,573 (19.6)
Hispanic	7,312 (55.9)
**Age group, y**	
50–64	10,941 (83.7)
65–75	2,138 (16.3)
**Sex**	
Male	5,878 (44.9)
Female	7,201 (55.1)
**Health insurance status**	
Medicare	1,173 (9.0)
Medicaid	1,958 (15.0)
Private	2,757 (21.1)
Medical access program	6,873 (52.6)
Grants	318 (2.4)
**Number of primary care–related visits in 12 months**	
0	2,622 (20.1)
1 or 2	3,960 (30.3)
3 or 4	3,146 (24.1)
>5	3,351 (25.6)
**Spatial access to medical home**	
Very close (<5 miles)	6,856 (52.4)
Close (>5 miles to <10 miles)	4,141 (31.7)
Far (>10 miles to <20 miles)	1,945 (14.9)
Very far (>20 miles)	137 (1.1)
**Spatial access to Dell Seton Medical Center**	
Very close (<5 miles)	2,592 (19.8)
Close (>5 miles to <10 miles)	6,193 (47.4)
Far (>10 miles to <20 miles)	3,865 (29.6)
Very far (>20 miles)	429 (3.3)

### Bivariate analysis

Distance of more than 20 miles to the offices of a primary care physician was negatively associated with CRC screening uptake (OR, 0.63; 95% CI, 0.43−0.93); we found similar results for distance to DSMC endoscopic services (OR, 0.80; 95% CI, 0.64−1.00). The number of primary care–related visits in 1 year was the strongest factor associated with up-to-date screening. Hispanic patients were more likely to be up to date than non-Hispanic White or African American patients, and women more likely to be screened than men. Patients aged 65 to 75 were more likely to keep up-to-date with screening than those aged 50 to 64. Patients supported financially by the county MAP or other grants had higher up-to-date screening rates, compared with those receiving benefits from Medicare, Medicaid, or private insurance (Table 2).

**Table 2:** Patient Screening Status (N = 13,079) and Unadjusted Odds Ratios of Up-to-Date Screenings, Central Texas, 2018

Variable	Screened (%)	Unscreened (%)	OR (95% CI)
**Race/ethnicity**			
Non-Hispanic White	970 (30.4)	2,224 (69.6)	1 [Reference]
Non-Hispanic African American	800 (31.1)	1,773 (68.9)	1.03 (0.92−1.16)
Hispanic	2,662 (36.4)	4,650 (63.6)	1.31 (1.20−1.44)^a^
**Age group, y**			
50–64	3,640 (33.3)	7,301 (66.7)	1 [Reference]
65–75	792 (37.0)	1,346 (63.0)	1.18 (1.07−1.30)^a^
**Sex**			
Male	1,736 (29.5)	4,142 (70.5)	1 [Reference]
Female	2,696 (37.4)	4,505 (62.6)	1.43 (1.33−1.54)^a^
**Health insurance status**			
Medicare	329 (28.1)	844 (72.0)	1 [Reference]
Medicaid	535 (27.3)	1,423 (72.7)	0.96 (0.82−1.13)
Private	775 (28.1)	1,982 (71.9)	1.00 (0.86−1.17)
Medical Access Program	2,649 (38.5)	4,224 (61.5)	1.61 (1.40−1.84)^a^
Grants for health care	144 (45.3)	174 (54.7)	2.12 (1.65−2.74)^a^
**Number of primary care–related visits in 1 y**			
0	337 (12.9)	2,285 (87.2)	1 [Reference]
1 or 2	1,107 (28.0)	2,853 (72.1)	2.63 (2.30−3.01)^a^
3 or 4	1,298 (41.3)	1,848 (58.7)	4.76 (4.16−5.45)^a^
≥5	1,690 (50.4)	1,661 (49.6)	6.90 (6.04−7.88)^a^
**Spatial access to medical home**			
Very close (≤5 miles)	2,359 (34.4)	4,497 (65.6)	1 [Reference]
Close (>5 miles to ≤10 miles)	1,388 (33.5)	2,753 (66.5)	0.96 (0.89−1.04)
Far (>10 miles to ≤20 miles)	651 (33.5)	1,294 (66.5)	0.96 (0.86−1.07)
Very far (>20 miles)	34 (24.8)	103 (75.2)	0.63 (0.43−0.93)^b^
**Spatial access to Dell Seton Medical Center**			
Very close (≤5 miles)	855 (33.0)	1,737 (67.0)	1 [Reference]
Close (>5 miles to ≤10 miles)	2,114 (34.1)	4,079 (65.9)	1.05 (0.96−1.16)
Far (>10 miles to ≤20 miles)	1,342 (34.7)	2,523 (65.3)	1.08 (0.97−1.20)
Very far (>20 miles)	121 (28.2)	308 (71.8)	0.80 (0.64−1.00)^b^

a *P* <.001.

b *P* <.05.

### Multivariate analysis

Effects of residing more than 20 miles away from a patient’s medical home or DSMC endoscopic services were no longer significant after adjustment for other variables. The association between up-to-date screening and each of the other variables was almost unchanged after adjustment. The number of primary care–related visits significantly influenced CRC screening, even after adjustment for race, ethnicity, age, sex, health insurance status, and spatial access to care (Table 3).

**Table 3:** Adjusted Odds Ratios (aORs) of Up-to-Date Screening of 13,079 Patients, Central Texas, 2018

Variables	aOR (95% CI)
**Race/ethnicity**	
Non-Hispanic White	1 [Reference]
African American	0.58 (0.39−0.87)^b^
Hispanics	0.94 (0.64−1.40)
**Ages, y**	
50−64	1 [Reference]
65−75	1.24 (1.11−1.38)^a^
**Sex**	
Male	1 [Reference]
Female	1.24 (1.15−1.35)^a^
**Health insurance status**	
Medicare	1 [Reference]
Medicaid	0.98 (0.82−1.16)
Private	1.30 (0.85,1.99)
Medical access program (MAP)	1.98 (1.70−2.31)^a^
Grants for health care	1.80 (1.12−2.89)^b^
**Number of primary care–related visits in 1 y**	
0	1 [Reference]
1 or 2	2.67 (2.34−3.06)^a^
3 or 4	4.68 (4.08−5.36)^a^
≥5	6.72 (5.87−7.70)^a^
**Spatial access to medical home**	
Very close (≤5 miles)	1 [Reference]
Close (>5 miles to ≤10 miles)	1.00 (0.91−1.09)
Far (>10 miles to ≤20 miles)	0.96 (0.85−1.08)
Very far (>20 miles)	0.79 (0.50−1.24)
**Spatial access to Dell Seton Medical Center**	
Very close (≤5 miles)	1 [Reference]
Close (>5 miles to ≤10 miles)	1.04 (0.93−1.15)
Far (>10 miles to ≤20 miles)	0.98 (0.87−1.10)
Very far (>20 miles)	0.82 (0.63−1.08)

a *P* <.001.

b *P* <.05.

### Geographic concentration of patients without up-to-date CRC screening

In the cluster analysis, only the clusters for all 21,923 patients combined were significant. We detected no significant clusters for either Hispanic patients or African American patients alone. For all patients combined, we found a cluster without up-to-date CRC screening that covered a large urban area located slightly toward the southwest part of urban Austin (Figure). 

**Figure:**
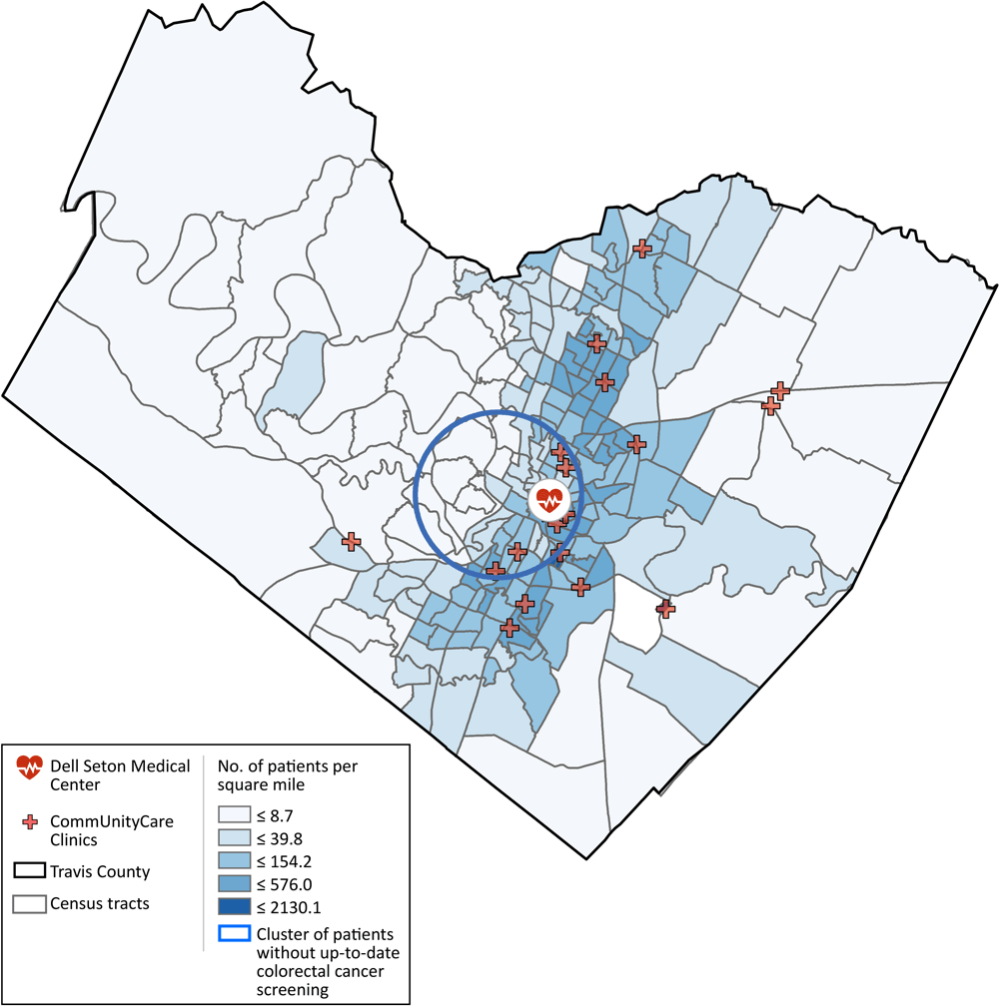
Medical facilities serving patients in Travis County, Texas and density of geocoded patients per square kilometer at the census tract level. A cluster shows levels of colorectal cancer screening was significantly lower, relative to patients from areas outside of the cluster but also served by the system of federally qualified health centers in the county. Radius of the circle is 4.0 miles.

The other significant cluster covered a smaller area southeast of the study area, where a correctional facility was located. Although the clusters associated with Hispanic and African American patients were not significant, they provided information about priority areas for interventions designed for these populations. Overall, the analysis identified areas for targeted intervention among the patients.

## Discussion

In a population of patients served by a large FQHC system, we found that residing more than 20 miles of driving distance from a primary care clinic was associated with low screening rates, and having more primary care visits within 1 year was associated with higher rates in unadjusted analyses. Driving distance, however, was not associated with screening after we adjusted for all covariates. The number of primary care visits remained a key factor after multivariate adjustment, suggesting that both access and use of care are key factors that affect screening for patients in this system. Overall screening rates in the system were generally low, a finding similar for other FQHC systems (14–16). Interventions that seek to increase screening in ways that do not require in-person visits or extended travel, such as mailed stool testing programs, may be effective in overcoming barriers (4,5,17).

Spatial analysis identified an area where the level of CRC screening was particularly low among the study population. These findings suggest the importance of identifying cluster area variations, engaging patients and providers, and increasing access for those who reside far from sources of care. The identified cluster area provides information about specific, localized needs for a geographically targeted intervention; however, additional data and community-engaged research are needed to examine factors associated with lower, up-to-date screening rates.

A large body of literature is available about CRC prevention, CRC screening, late-stage diagnosis, cancer mortality, and disparities (17–26). Our search for this study, however, found only 2 studies in the United States that examined the association between CRC screening status and travel time to care (21,22). One study found no association between travel time and the likelihood of metastatic cancer in an insured population, but it did find an association between previous use of preventive care and the likelihood of metastatic cancer (21). The other study examined multiple factors associated with screening in patients at the Bellevue Hospital system in New York City. That study found no association between screening and travel time among patients who had at least one clinic visit; however, more primary care visits were positively associated with screening (22). In contrast, our study indicates that driving distance to care more than 20 miles is negatively associated with CRC screening uptake. We found that a large number of primary care visits within 1 year was significantly associated with a high rate of up-to-date CRC screening. This finding echoes the literature (21–24) and suggests that more primary care–related visits increase opportunities for screening.

Our study has several limitations. First, nearly 20% of the records in the original CommUnityCare patient database had either incomplete, insufficient, or incorrect address information, which that made it impossible to achieve a high rate of geocoding. Second, we had to exclude more than 40% of the geocoded patient records in our logistic regression analysis involving both aspatial and spatial data because of incorrect or insufficient information. Results from the larger set of 27,285 patients, with only aspatial variables in the supplemental analyses, however, confirmed results reported in this study.

Third, many patients use public transportation to reach a clinic. This mode of transportation is different from using an automobile. Thus, the use of driving distance as a measure of spatial access might underestimate the challenges some patients face in accessing care, and we did not have individual-level data for transportation access to better explore this phenomenon. Fourth, we had to rely on a limited number of covariables available from administrative data sets. We hope to extend our analysis with additional clinical and behavioral variables in future research.

Fifth, the overall CRC screening rate in the study population was low, even in comparison with rates of CRC screening among other FQHC patients. This low rate suggests that opportunistic efforts alone have been ineffective and may be a result of competing health care demands, including preventing and treating other chronic conditions and the lack of a preventive care reminders in the FQHC’s electronic health record system. Associations identified here may differ in other populations, including other groups of disproportionately affected patients who have higher levels of screening (15). Finally, our study examined patients in an urban FQHC system in a county that offers a medical assistance program. The factors affecting screening are likely to be different for people who do not have a regular source of care, for those who reside in rural areas, or those who do not have access to preventive care.

Based on data about patients served by an urban FQHC system in Central Texas, our study achieved its objectives. We found that regular visits for primary care are positively associated with up-to-date CRC screening, and residing greater than 20 miles of driving distance to care providers is negatively associated with CRC screening uptake. Additionally, our study detected that patients in the southwest area of urban Austin, Texas, have a significantly low rate of up-to-date CRC screening. The analyses provide valuable insights to support targeted interventions to increase screening, both for our FQHC system and others. We prioritize interventions that identify unscreened patients apart from opportunistic visit-based care, inform patients about their eligibility for screening, and invite them into care. Mailed fecal immunochemical test programs are particularly effective and efficient (4,5,17) and are the principal intervention in our system to increase CRC screening, coupled with patient navigation to help identify and reduce transportation barriers. We plan to adopt a new electronic health record that includes preventive care prompting and to conduct additional formative work to understand barriers for patients who do not respond to the interventions.
